# Linear Morphometry of Male Genitalia Distinguishes the Ant Genera *Monomorium* and *Syllophopsis* (Hymenoptera: Formicidae) in Madagascar

**DOI:** 10.3390/insects15080605

**Published:** 2024-08-11

**Authors:** Nomena F. Rasoarimalala, Tanjona Ramiadantsoa, Jean Claude Rakotonirina, Brian L. Fisher

**Affiliations:** 1 Madagascar Biodiversity Center, Parc Botanique et Zoologique de Tsimbazaza, Antananarivo 101, Madagascar; ramiadantsoa@wisc.edu (T.R.); jcrakoto25@gmail.com (J.C.R.); bfisher@calacademy.org (B.L.F.); 2 Mention Entomologie Cultures Élevage et Santé, Faculté des Sciences, Université d’Antananarivo, Antananarivo 101, Madagascar; 3 Department of Entomology, California Academy of Sciences, San Francisco, CA 94118, USA

**Keywords:** species delimitation, male genitalia, linear morphometry, multivariate analysis, Formicidae, Madagascar

## Abstract

**Simple Summary:**

We evaluated linear morphometry of male genitalia as a diagnostic method to distinguish the genera and species of *Monomorium* and *Syllophopsis* (Hymenoptera: Formicidae). We measured 10 morphometric characters on the male genitalia from 10 species of *Monomorium* and 5 species of *Syllophopsis*. We used three datasets, raw data, ratio data, and RAV data, and analyzed them using multivariate methods: hierarchical clustering (Ward’s method), Principal Component Analysis (PCA), Non-Metric Multidimensional Scaling analyses (NMDS), Linear Discriminant Analysis (LDA), and Conditional Inference Trees (CITs). The ratio data were most effective in separating the two genera, while the raw data were more effective at species-level delimitation. The findings highlighted the potential for a broader application of genitalia-based morphometric analyses in ant systematics.

**Abstract:**

Morphometric analyses of male genitalia are routinely used to distinguish genera and species in beetles, butterflies, and flies, but are rarely used in ants, where most morphometric analyses focus on the external morphology of the worker caste. In this work, we performed linear morphometric analysis of the male genitalia to distinguish *Monomorium* and *Syllophopsis* in Madagascar. For 80 specimens, we measured 10 morphometric characters, especially on the paramere, volsella, and penisvalvae. Three datasets were made from linear measurements: mean (raw data), the ratios of characters (ratio data), and the Removal of Allometric Variance (RAV data). The following quantitative methods were applied to these datasets: hierarchical clustering (Ward’s method), unconstrained ordination methods including Principal Component Analysis (PCA), Non-Metric Multidimensional Scaling analyses (NMDS), Linear Discriminant Analysis (LDA), and Conditional Inference Trees (CITs). The results from statistical analysis show that the ratios proved to be the most effective approach for genus-level differentiation. However, the RAV method exhibited overlap between the genera. Meanwhile, the raw data facilitated more nuanced distinctions at the species level compared with the ratios and RAV approaches. The CITs revealed that the ratios of denticle length of the valviceps (SeL) to the paramere height (PaH) effectively distinguished between genera and identified key variables for species-level differentiation. Overall, this study shows that linear morphometric analysis of male genitalia is a useful data source for taxonomic delimitation.

## 1. Introduction

Examining characters of male genitalia is a useful diagnostic method in insect systematics [1,2,3]. Differences in genitalia morphology are conspicuous as the characters rapidly and independently evolve and diverge to reinforce reproductive isolation via sexual selection [4]. For instance, in dung beetles, the pygidium and aedeagus of the genitalia have evolved 5.5 times faster than the foretibia [5]. Genitalia characters are more reliable for determining evolutionary relationships than other non-genitalia characters [6,7]. External characters, i.e., body shape and color, can appear similar across different species due to convergent evolution, and individual variations within a species. However, genitalia are less affected by these issues, making them useful for distinguishing between species.

Species description and identification have historically been based on a heuristic approach, where scientists rely on their ability to identify the morphological singularity of species. However, finding qualitative differences that align with the discrete nature of a species is not always possible. This is because species are complex biological entities, and their boundaries can be blurred or overlapping, making it challenging to define clear-cut distinctions between them. With methodological advances, including Scanning Electron Microscopy (SEM) [8] and Micro X-ray Computed Tomography (μCT) [9,10,11], morphometric methods such as linear morphometry [12,13] and geometric morphometry [14,15,16] are now routinely included in systematic studies. Beyond species descriptions, data are also used to delimit/classify species via algorithms and statistical methods. Raw or transformed data (e.g., ratios or log-transformed) [17,18] are used in conjunction with multivariate methods to classify species using methods that range from simple hierarchical clustering [19], to Elliptic Fourier Analysis [20], and advanced machine learning [21]. Congruence among different methods provides stronger evidence of species classification [22,23,24].

Morphometric analyses of male genitalia are commonly used in various insect orders, such as Coleoptera [5,6,25], Lepidoptera [12,26,27,28,29], Hemiptera [20,30,31,32,33,34], and Diptera [35]. In ants, however, morphometric analyses of the male genitalia are relatively rare, and studies primarily focus on qualitative descriptions [9,36,37,38,39,40,41,42,43,44]. To date, only two studies have carried out quantitative morphometric analyses of the male genitalia, namely the genus *Dinoponera* [16] and the genus *Tapinoma* [45]. In contrast to the few studies involving morphometry of male genitalia, hundreds of studies have used discriminant analysis of worker caste to differentiate ant species [17,46,47,48,49,50,51]. These studies have utilized multivariate methods such as hierarchical clustering (Ward’s method or UPGMA), non-hierarchical clustering (K-means partitioning), and other unconstrained ordinations (PCA, NMDS). These multivariate methods, combined with data transformations, significantly enhance species discrimination. The Removal of Allometric Variance (RAV) is a common data transformation that involves performing a linear regression on the ratios of characters [52]. This method helps to eliminate the effects of size differences and focuses on the shape and proportions of the morphological features [17]. This approach ensures that size variations do not bias the analysis and provides a more accurate representation of the morphological differences between ant species. 

In this work, we evaluate the application of linear morphometric analysis of male genitalia to distinguish two ant genera, *Monomorium* and *Syllophopsis*, in Madagascar. We chose to focus our study on these two genera because recent molecular phylogenetic evidence [53] has demonstrated that *Syllophopsis* represents a distinct evolutionary lineage that was previously synonymized under *Monomorium* [54,55]. This reclassification highlights the need for a more thorough investigation of the diagnostic characters, particularly in the males, which are the focus of our current work. Although the two genera are phylogenetically distinct, limited morphological information is available to distinguish the males of each genus [56,57]. Although the existing keys by Ramamonjisoa et al. [56,57] allows for reliable identification, applying morphometric analysis can provide additional insights into the morphological differentiation and taxonomic classification of these closely related genera. We measured ten characters on the genitalia of a total of 80 specimens, comprising 54 specimens of *Monomorium* and 26 specimens of *Syllophopsis*. We applied a combination of data transformation and four multivariate methods: hierarchical clustering (Ward’s method), and unconstrained ordination methods (PCA, NMDS, LDA) to distinguish *Monomorium* and *Syllophopsis* at the genus and species level. We also applied a Conditional Inference Trees (CITs) method [58] to identify key characters that could be used to separate them at the genus and species level. Most specimens used in this study were not associated with workers and could not be associated with named species in most cases. However, we could categorize them into morphospecies for both genera based on their morphological characteristics.

## 2. Materials and Methods

### 2.1. Data Source

This study included males from 14 locations across Madagascar (Table S1). The ants were collected using Malaise traps and were immediately preserved in 95% ETOH before being stored in vials. The specimens are a part of the Entomology collection at the Madagascar Biodiversity Center (MBC), Antananarivo, Madagascar. To identify the males, keys outlined by Ramamonjisoa et al. [56,57] were used to identify them at the genus level. However, owing to the absence of species-level keys and an in-depth study on the males of the genera *Monomorium* and *Syllophopsis* from Madagascar, specimens were categorized into morphospecies based on their morphology. Based on images of males from a nest series on Antweb (https://www.antweb.org/) (accessed on 1 March 2022), specimens were identified as a valid species (*M. madecassum*, *M. pharaonis*, *M. hanneli*, *S. fisheri*, *S. modesta*), or identified to a morphospecies that is similar to valid species (M. termitobium_nr01, M termitobium_nr02, S. hildebrandti_nr01, S. hildebrandti_nr02); or to a morphospecies distinct from known species (*M*. MG01/02/03, *S*. MG01).

### 2.2. Slide Preparation and Measurements

**Figure 1:**
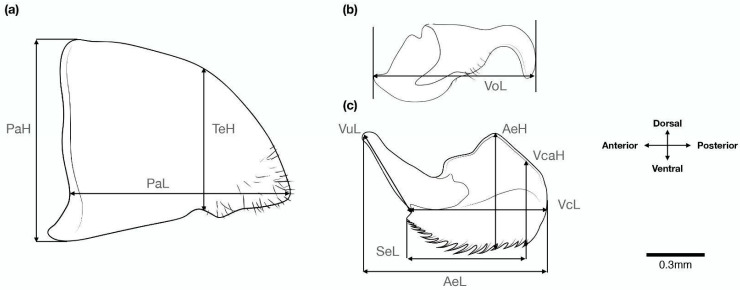
Illustration of the linear measurements applied to the (**a**) paramere, (**b**) volsella, and (**c**) penisvalvae taken from *Monomorium madecassum*. Illustrations by the author.

The genitalia of 80 male specimens belonging to two genera, *Monomorium* (54) and *Syllophopsis* (26), were used in this study (Table S1). Genitalia preparation followed the method outlined in Tozetto and Lattke [16]. The paramere, volsella, and penisvalvae were mounted on glycerol jelly between microscope slides and cover slips, labeled, and digitized with a Lumascope LS520. Using the ImageJ 1.34 Java image processing program (US National Institutes of Health: http://imagej.nih.gov/ij/) (accessed on 15 May 2022) [59], ten characters (Figure 1) were measured by assessing the distance between landmarks after calibration. The characters were selected to measure the height and length of components of the paramere, volsella, and penisvalvae. Explanations and abbreviations for measured characters are given in Table 1 and illustrated in Figure 1. The terminology adopted for male genitalia follows Boudinot [43]. 

### 2.3. Morphometric Data

We performed each measurement four times at the same point for every specimen. Subsequently, we applied the Intraclass Correlation Coefficient (ICC) from the “irr” package in R [60] to evaluate the reliability of the four measurements. It should be noted that ICC indicates excellent reliability when its value gets closer to 1 [61] (Table S2). The raw data were obtained by taking the mean of the four measurements (Table 2). We generated the ratio data from the raw data by dividing the characters by the height of the paramere (PaH). We opted for PaH instead of the standard measure for body size, mesosoma length (also known as Weber’s length) as we did not take body measurements. We created a third dataset by performing the Removal of Allometric Variance on the characters using the height of the paramere (PaH) in the denominator. RAV is essentially a regression on the ratios of the characters (Table S3) (for more details see [17,45,51,52]). Hereon, the third dataset is called RAV data.

### 2.4. Data Analyses

For each type of data, we constructed a distance matrix based on Euclidean distance and then performed hierarchical clustering (Ward’s method), and ordination methods (PCA, NMDS, and LDA). For the ordination methods, we only focused on the first two axes. Ward’s method was conducted to group specimens based on similarity, helping to identify natural groupings and relationships that may correspond to species boundaries [62]. PCA was employed for dimensionality reduction while preserving variance, making it useful for exploratory data analysis [63]. We also used NMDS to visualizes similarities among data points based on rank-ordering distances [64]. Additionally, LDA was applied as a classification technique to assess how well morphometric traits distinguish between different species or groups [65]. Lastly, we applied the Conditional Inference Trees (CITs) method [58] using the package “partkyt” [66] to identify characters that could be used to separate the genus and the species. All statistical analyses were carried out in R [67]. 

## 3. Results

### 3.1. Multivariate Analysis of Morphometric Data

#### 3.1.1. Raw Data

**Figure 2:**
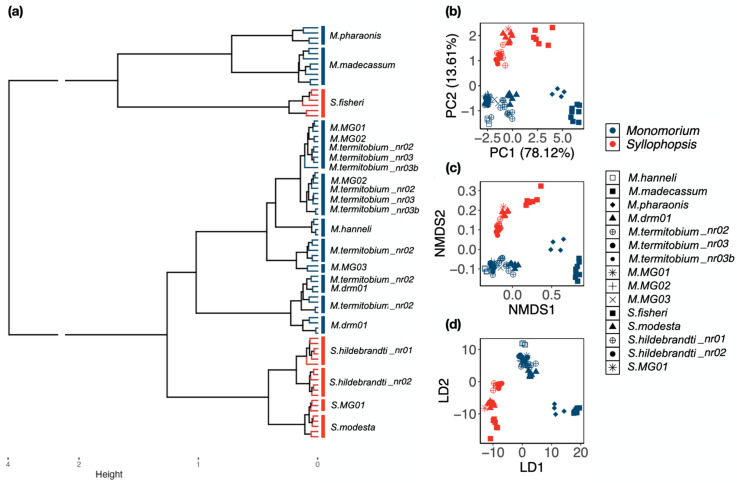
Classification at the genus and species levels based on the raw data using (**a**) Ward’s method, (**b**) Principal Component Analysis, (**c**) Non-Metric Multidimensional Scaling, and (**d**) Linear Discriminant Analysis. (**a**) *Monomorium* is depicted in blue, and *Syllophopsis* in red. In (**b**–**d**), squares and diamonds represent valid species, and round shapes represent morphospecies that are similar to valid species. Crosses and asterisks represent morphospecies distinct from known species.

Ward’s method: *Monomorium* and *Syllophopsis* did not form two distinct groups in the hierarchical clustering (Figure 2a). There were four major groups: a group composed of *M. madecassum* and *M. pharaonis*, a group composed of *S. fisheri*, a group composed of the non-*fisheri Syllophopsis*, and a group composed of the remaining *Monomorium*. At the species level, the four distinct species of *Monomorium* were *M. madecassum*, *M. pharaonis*, *M. hanneli*, and *M*. MG03, and all five for *Syllophopsis*: *S. fisheri*, *S. hildebrandti_*nr01, *S. hildebrandti_n*r02, *S. modesta*, and *S*. MG01 (Figure 2a). 

PCA, NMDS, LDA: The three ordination methods gave qualitatively similar results. *Monomorium* and *Syllophopsis* were clearly separated (Figure 2b–d). This method revealed six groups: *M. madecassum*, *M. pharaonis*, *M. hanneli*, *S. fisheri*, the remaining *Monomorium*, and the remaining (non-*fisheri*) *Syllophopsis* (Figure 2b–d). For the remaining *Monomorium* group, there seemed to be a continuum. *M. termitobium_*nr03/03b (filled circles) and *M. drm01* (triangle) could be separated if *M. termitobium_*nr02 (non-filled circle), *M*. MG01/02/03 (non-filled symbols) were excluded (Figure 2b–d). 

The same pattern was also found for non-*fisheri Syllophopsis*: *S. modesta* (triangle) and *S. hildebrandti_*nr02 (filled circle) were distinct without *S. hildebrandti_*nr01 (non-filled circle) and *S*. MG01 (non-filled symbol) (Figure 2b–d). In the LDA, the *S. hildebrandti_*nr01/02 groups, the *S. modesta* group, and the *S*. MG01 group were almost distinct (Figure 2d). The representation in two dimensions in the PCA and NMDS captured most of the variation; the percentage of variance explained was high (91.73%) in the PCA, and stress was low (0.016) in the NMDS. 

#### 3.1.2. Ratio Data

**Figure 3:**
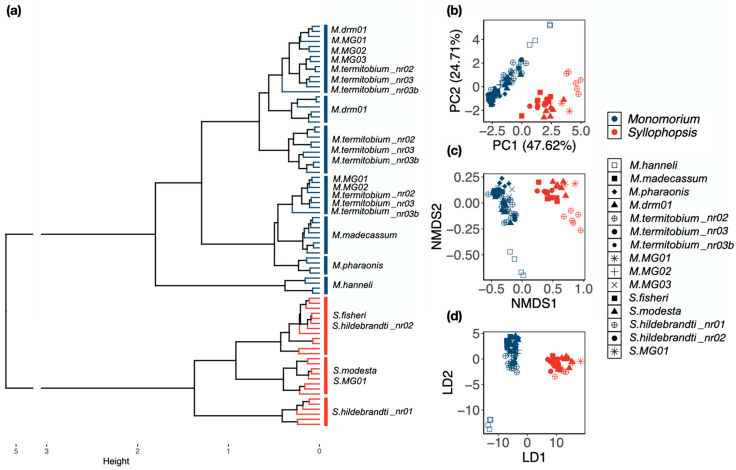
Classification at the genus and species level based on the ratio data using (**a**) Ward’s method, (**b**) Principal Component Analysis, (**c**) Non-Metric Multidimensional Scaling, and (**d**) Linear Discriminant Analysis. (**a**) *Monomorium* is depicted in blue, and *Syllophopsis* in red. In (**b**–**d**), squares and diamonds represent valid species, and round shapes represent morphospecies that are similar to valid species. Crosses and asterisks represent morphospecies distinct from known species.

Ward’s method: *Monomorium* and *Syllophopsis* were separated into two distinct groups in the hierarchical clustering (Figure 3a). There were three groups among *Syllophopsis*: *S. hildebrandti_*nr01, *S. fisheri*; *S. hildebrandti_*nr02; and *S. modesta* and *S*. MG01. Among *Monomorium*, species were distributed across different groups. At the species level, the distinct groups were *M. madecassum*, *M. pharaonis*, and *M. hanneli*, *S. hildebrandti_*nr01 (Figure 3a). Remarkably, *M. hanneli* appeared as a distinct group between *Monomorium* and *Syllophopsis* species (Figure 3a).

**PCA, NMDS, LDA:** The two genera were clearly separated in the three ordination methods (Figure 3b–d). The first axis sufficed to separate both genera in the NMDS and LDA (Figure 3c–d). At the species level, unlike the raw data, there were no clear groupings and the results varied with the methods. All *Monomorium* overlapped in the PCA, NMDS, and LDA except *M. hanneli* (Figure 3c,d). For *Syllophopsis*, only *S. hildebrandti_*nr01 stood out in the PCA and NMDS (Figure 3b,c). The LDA showed that all the species of *Syllophopsis* overlapped (Figure 3d). The percentage of variance explained was high (72.33%) in the PCA and stress was low (0.044) in the NMDS. 

#### 3.1.3. RAV Data (Removal Allometric Variance)

**Figure 4:**
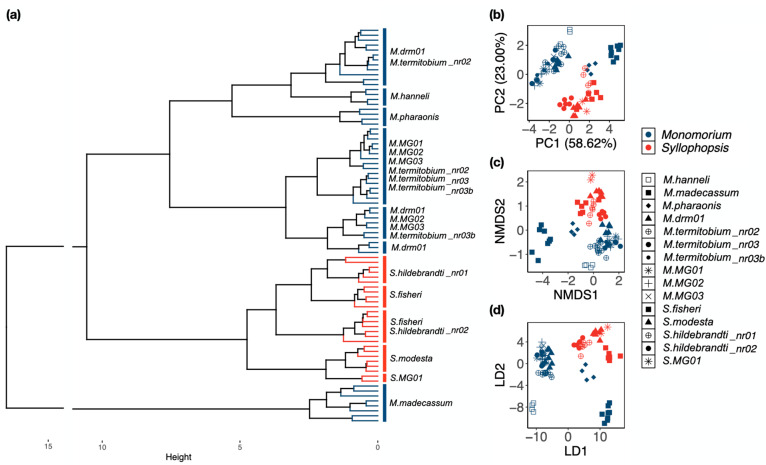
Classification at the genus and species level after the effect of allometric variance was removed (RAV data) using (**a**) Ward’s method, (**b**) Principal Component Analysis, (**c**) Non-Metric Multidimensional Scaling, and (**d**) Linear Discriminant Analysis. (**a**) *Monomorium* is depicted in blue, and *Syllophopsis* in red. In (**b**–**d**), squares and diamonds represent valid species, and round shapes represent morphospecies that are similar to valid species. Crosses and asterisks represent morphospecies distinct from known species.

**Ward’s method:***Monomorium* and *Syllophopsis* did not form two distinct groups in the hierarchical clustering (Figure 4a). *Syllophopsis* was squeezed between two groups of *Monomorium. M. madecassum* was expelled from the *Monomorium*-group. The remaining *Monomorium* formed a distinct group. At the species level, the distinct groups were *M. madecassum*, *M. pharaonis*, *M. hanneli*, *S. hildebrandti_*nr01, *S. modesta*, and *S*. MG01 (Figure 4a).

**PCA, NMDS, LDA:** Similar to the hierarchical clustering results, *Monomorium* and *Syllophopsis* overlapped in the ordination methods (Figure 4b–d). *M. madecassum* and *M. pharaonis* bridged three separate groups—the encroachment was most apparent in the PCA (Figure 4b). At the species level, the methods yielded different results. For instance, *M. madecassum*, *S. modesta*, and *S. hildebrandti_*nr02 stood out in the PCA (Figure 4b); *M. madecassum*, *M. pharaonis*, *M. hanneli*, *S*. MG01, and *S. hildebrandti_*nr02 in NMDS (Figure 4c); and *M. madecassum*, *M. pharaonis*, *M. hanneli*, and *S. fisheri* in the LDA (Figure 4d). The percentage of variance explained was 81.62% in the PCA, and stress was high (0.071) in the NMDS.

### 3.2. Conditional Inference Trees (CITs)

#### 3.2.1. Raw Data

**Figure 5:**
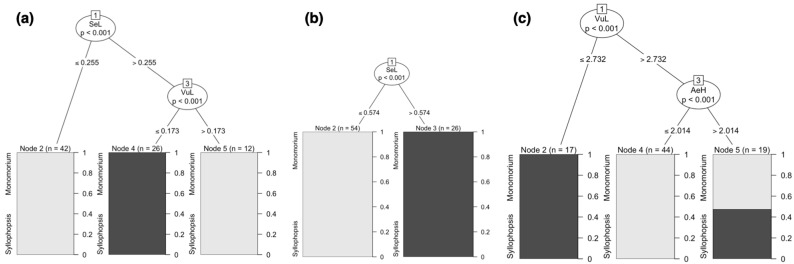
Conditional Inference Trees based on (**a**) raw data, (**b**) ratio data, and (**c**) RAV data were calculated. *Monomorium* is in light gray, and *Syllophopsis* is in dark gray. Each node represents a morphometric trait used for classification. Terminal nodes display the proportion of specimens classified into each group, with sample sizes (N) provided.

**Figure 6:**
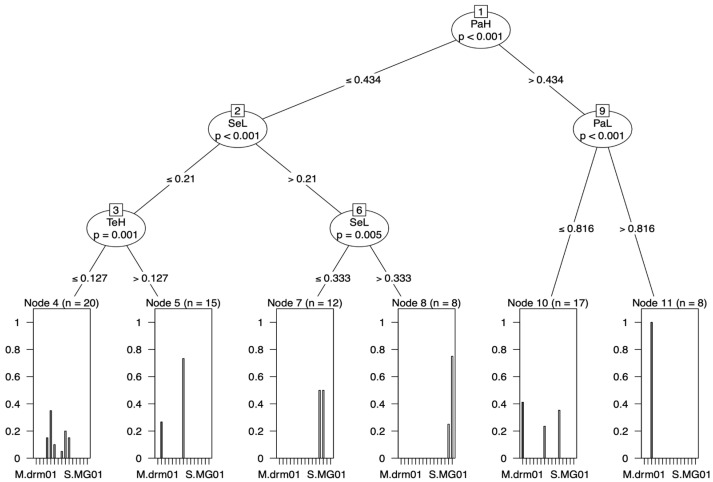
Classification tree from the conditional inference trees (CITs) model (raw data). Each node represents a morphometric trait used for classification. Terminal nodes display the proportion of specimens classified into each species, with sample sizes (N) provided.

At the genus level, CITs identified two characters to separate *Syllophopsis* and *Monomorium:* the length of the valviceps denticles (SeL) and the length of the valvura (VuL). *Syllophopsis* was distinguished from *Monomorium* by the length of the valviceps denticles (SeL) > 0.255 and the length of the valvura (VuL) ≤ 0.173 (Figure 5a). At the species level, *M. madecassum* was distinguished using pairs of characters with PaH > 0.434 and PaL > 0.816 (Figure 6).

#### 3.2.2. Ratio Data

**Figure 7:**
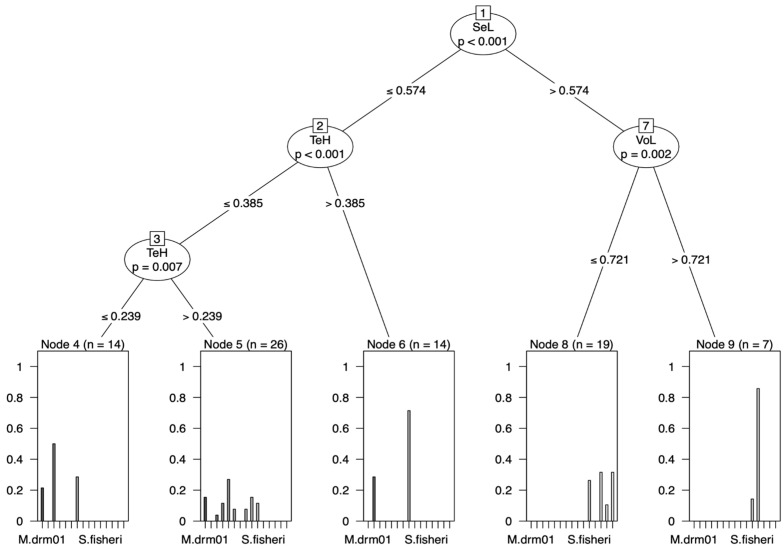
Classification tree from the conditional inference trees (CITs) model (ratio data). Each node represents a morphometric trait used for classification. Terminal nodes display the proportion of specimens classified into each species, with sample sizes (N) provided.

At the genus level, one character could distinguish the two genera. *Syllophopsis* was distinguished from *Monomorium* by the ratios of the valviceps dentition (SeL) and the height of the paramere (PaH) > 0.574 (Figure 5b). At the species level, none of the other species of *Monomorium* and *Syllophopsis* exhibited a distinct separation (Figure 7).

#### 3.2.3. RAV Data

**Figure 8:**
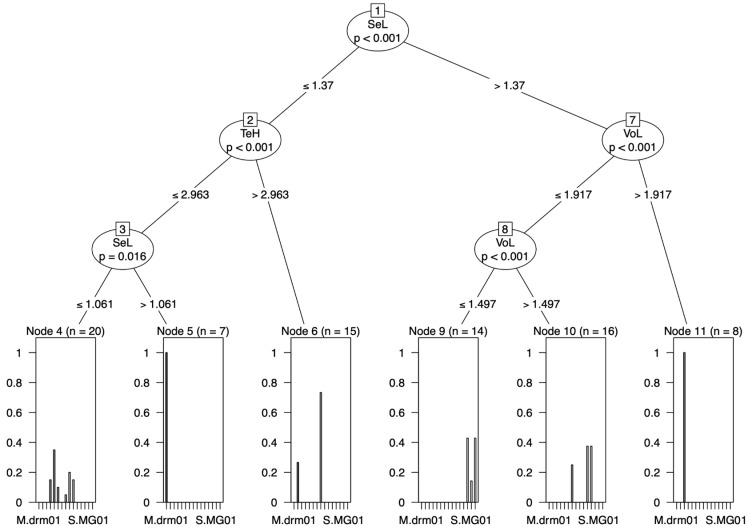
Classification tree from the conditional inference trees (CITs) model (RAV data). Each node represents a morphometric trait used for classification. Terminal nodes display the proportion of specimens classified into each species, with sample sizes (N) provided.

At the genus level, the results of the CITs were qualitatively similar to those of Ward’s method (Figure 4a). *Syllophopsis* overlapped in the RAV data (Figure 5c). However, two characters were chosen by CITs: the valvura (VuL) and valviceps height (AeH). At the species level, two species showed a clear split: *M. madecassum* was distinguished with SeL > 1.37 and VoL > 1.917 and *M. drm01* with TeH ≤ 2.963 and SeL > 1.061 (Figure 8). 

## 4. Discussion

### 4.1. Multivariate Analysis of Morphometric Data

At the genus level, the raw and ratio data datasets analyzed with all methods distinguished *Monomorium* and *Syllophopsis* in Madagascar. Still, the ratio data were the only dataset to separate *Monomorium* and *Syllophopsis* into two distinct groups, since the ratio data performed better than the raw data at separating genera. This result suggests that shape, rather than size, is more important in the evolution of reproductive systems in these genera.

The raw and RAV data did not isolate two distinct clusters corresponding to *Monomorium* and *Syllophopsis*. For the raw data, the “culprits” were *M. madecassum*, *M. pharaonis*, and *S. fisheri* which grouped together and not with their congeners (Figure 2a). These three species are the largest, which may explain why they form a cluster with the raw data since raw data preserve size. For the RAV data, the “culprit” was *M. madecassum* which grouped next to the *Syllophopsis* cluster (Figure 4a). The exclusion of *M. madecassum* from the *Monomorium* group in the RAV data and the consequent overlap of the genera remains unclear, warranting further investigation into the morphometric characteristics that distinguish this species from its congeners.

At the species level, the raw data allowed us to distinguish the greatest number of species among the three datasets. Using Ward’s method, four out of ten species of *Monomorium* appeared distinct: *M. madecassum*, *M. pharaonis*, *M. hanneli*, and *M*. MG03. Likewise, all five species of *Syllophopsis* were distinguishable (Figure 2a). The three ordination methods gave qualitatively similar results: *M. madecassum*, *M. pharaonis*, *M. hanneli*, and *S. fisheri* were distinct. Notably, the first three species are the largest in body size, with *M. hanneli* being the smallest, considering the correlation between genitalia and ant body size. This observation may imply their isolation. 

The outcomes varied across the methods for the ratio data compared to the raw data (Figure 3a–d). Using Ward’s method, four species could be identified: *M. madecassum*, *M. pharaonis*, *M. hanneli*, and *S. hildebrandti_*nr01 (Figure 3a). All *Monomorium* species exhibited overlap in the PCA, NMDS, and LDA plots, except for *M. hanneli* (Figure 3c,d). The distinction of *M. hanneli* aligns with previous molecular phylogenetic studies indicating that *M. hanneli* is genetically distinct from other *Monomorium* species and may not belong to the genus [53]. Among *Syllophopsis*, only *S. hildebrandti_*nr01 stood out in the PCA and NMDS plots (Figure 3b,c), while the LDA revealed overlap among all *Syllophopsis* species (Figure 3d).

Although there is an overlap between the species of the two genera (Figure 4b,c), the RAV data could still isolate more species compared to the ratio data. In contrast to the raw data, the RAV data outcome differed across methods. Ward’s method identified *M. madecassum*, *M. pharaonis*, *M. hanneli*, *S. hildebrandti_*nr01, *S. modesta*, and *S*. MG01 as distinct species (Figure 4a). PCA distinguished *M. madecassum*, *S. modesta*, and *S. hildebrandti_*nr02 (Figure 4b). The NMDS plot highlighted *M. madecassum*, *M. pharaonis*, *M. hanneli*, *S*. MG01, and *S. hildebrandti_*nr02 (Figure 4c). LDA separated *M. madecassum*, *M. pharaonis*, *M. hanneli*, and *S. fisheri* (Figure 4d). Interestingly, the LDA results for RAV data were similar to the ordination outcomes for raw data. By removing allometric variance, RAV allows LDA to better identify the shape differences that distinguish species, leading to improved discriminatory performance. 

In contrast to a prior study that successfully differentiated male species using RAV [45], our findings did not demonstrate significant discrimination among all species. We can only speculate on the reasons for this failure. On one hand, it could indicate methodological or data limitations, which we discuss extensively in the caveats section. On the other hand, our findings could reveal real patterns. For instance, the overlap among *M. termitobium*_nr02/03/03b and *M*. MG01/02, which were collected in the same area (Table S1), could indicate that they are not separate species.

### 4.2. Conditional Inference Trees (CITs)

At the genus level, the ratios of valviceps dentition and paramere height (SeL/PaH) had the most discriminative power (Figure 5b). The valviceps dentition (SeL) which is part of the penisvalvae refers to the tooth-like structures on the valviceps (Figure 1c). These toothlike structures are involved in the process of sperm transfer and can vary in shape and size between different ant species [16,68], making it important in species differentiation [9,44]. 

At the species level, one species was differentiated using the raw data, and two using the RAV data; however, no clear split was found using the ratio data (Figure 6, Figure 7 and Figure 8). In the raw data, *M. madecassum* was distinctly identified using two characters: PaH (paramere height) and PaL (paramere length). Using the RAV data, *M. madecassum* was distinguished by SeL (valviceps length) and VoL (volsella length), while *M. drm01* was differentiated by TeH (telomere length) and SeL (valviceps length) (Figure 8). These results indicate that *M. madecassum* and *M. drm01* exhibit unique morphological features that can be quantified to differentiate them from their counterparts. Although none of the species of *Monomorium* and *Syllophopsis* showed a clear split using ratio data, the CITs analysis provided a comprehensive and informative overview of the key morphological characteristics to prioritize when distinguishing among species. Overall, CITs proves particularly useful in studies involving numerous measured traits, enhancing accuracy and reliability.

### 4.3. Caveats

Overall, our goal was not to support or resolve current taxonomy, nor to describe new species. Rather, we wished to investigate the potential of linear morphometry of male genitalia to distinguish ant genera and species. Our study analyzed 80 male specimens, from 15 taxa, (Table S1). The large number of taxa with relatively few specimens per species suggested our model was underpowered and made the delimitation of all species likely to fail. In comparison, Seifert [51] used 213 specimens and 11 characters to separate two *Hypoponera* species. The use of paramere height (PaH) as a dependent variable in RAV calculations, instead of the standard measure for body size in ants, mesosoma length (also known as Weber’s length), may also have influenced the results. We relied on PaH as we did not take body measurements, which could be considered a limitation of the study. Future studies could assess how adding more specimens, characters, and species outside the Malagasy region would clarify or blur our classification. From a methodological perspective, additional statistical methods could be tested. For example, silhouette width [69] could be used to measure the robustness of the clusters or how many axes should be considered in the ordinations. For ants, including the nest centroid approach in conjunction with multivariate methods could improve discrimination [70] and procedures exist to analyze ratio data in conjunction with PCA and LDA [68]. 

Although morphometric analysis of genitalia has many advantages, considerable effort is involved in obtaining the data: males are rare, and specimens are usually disarticulated when preparing the measurements, a situation particularly common with the abdomen. These issues make it difficult to impossible to conduct further analyses or observations on the same specimen. However, it is worth considering the trade-offs between non-trivial data collection and the power/precision of delimitation. When precision is required to, for example, reveal cryptic species or traits due to environmental filtering, the study of genitalia could be advantageous. 

## 5. Conclusions

We explored the potential of linear morphometry of male genitalia to separate two similar genera, *Monomorium* and *Syllophopsis*, in Madagascar. The choice of data type, whether raw, ratio, or RAV, can significantly impact the ability to detect differences at different taxonomic levels. This observation highlights the importance of carefully considering the data type selection in morphometric studies, as it can have a profound influence on the research findings and conclusions.

The method used in this study: Ward’s method, PCA, NMDS, and LDA each have unique strengths and weaknesses. Ward’s method creates hierarchical clusters but is sensitive to outliers, PCA is good for dimensionality reduction but assumes linearity [71], while NMDS captures non-linear relationships [64], and LDA offers clear group distinctions but relies on normality assumptions [65]. Ultimately, while all methods yield similar results, we recommend using LDA as it provides clearer groupings (Figure 2d, Figure 3d and Figure 4d) and analyzing both raw data and ratios to maximize the effectiveness of genitalia morphometry in differentiating ant taxa in future studies.

Taking the ratios of the characters was the most efficient way to separate the genera. Such a result is robust to the clustering, ordination, and regression methods we used, and suggests that the shape of genitalia is important in the diversification of this group. Raw data, however, allowed us to distinguish more species and suggested that differences in size are more important for species-level differentiation. Our results are in line with the idea that the shape and size of genitalia matter when distinguishing species [16,72]. Linear morphometric analysis of male genitalia is a powerful tool to distinguish, stabilize, and accelerate taxonomic work. This quantitative approach (applying discriminant methods to continuous traits) complements the more traditional qualitative approaches (examination of morphological characters) and is of particular interest to researchers in developing countries with limited access to molecular analyses and advanced equipment. This integrative approach is particularly valuable in complex fields like taxonomy, where nuanced morphological differences can be critical for accurate species identification and classification.

## Figures and Tables

**Table 1:** Abbreviations of morphometric characters, definition of measurements.

Abbreviation: Description	Character Definition	Figure
PaH: paramere height	Maximum height of the paramere in lateral view	Figure 1a
PaL: paramere length	Maximum length of the paramere in lateral view	Figure 1a
TeH: telomere height	Maximum height of the telomere in lateral view	Figure 1a
VoL: volsella length	Distance from the border of the basivolsella to the apex of the digitus in lateral view	Figure 1b
AeH: valviceps height	Maximum height of the valviceps in lateral view	Figure 1c
AeL: penisvalvae length	Maximum length from the apex of the valviceps to the valvura in lateral view	Figure 1c
SeL: valviceps denticles length	Distance between the first basal denticle to the first apical denticle of valviceps in lateral view	Figure 1c
VcL: valviceps length	Distance between the apex of the valviceps, not including the valvura in lateral view	Figure 1c
VcaH: apical height of the valviceps	Assessed vertically to the first apical denticle of the valviceps in lateral view	Figure 1c
VuL: valvura length	Maximum length of the valvura in lateral view	Figure 1c

**Table 2:** Morphometric data of male genitalia of *Monomorium* and *Syllophopsis*. Numbers represent the mean ± standard deviation, and the range [minimum, maximum].

**Species**	**PaH**	**PaL**	**TeH**	**VoL**	**AeH**
*M*. MG01 (*n* = 3)	0.342 ± 0.015 [0.333, 0.359]	0.365 ± 0.016 [0.347, 0.376]	0.103 ± 0.014 [0.087, 0.111]	0.204, 0.016 [0.194, 0.222]	0.157 ± 0.004 [0.154, 0.162]
*M*. MG02 (*n* = 7)	0.344 ± 0.021 [0.311, 0.374]	0.357 ± 0.021 [0.335, 0.387]	0.104 ± 0.012 [0.09, 0.118]	0.188, 0.013 [0.167, 0.207]	0.149 ± 0.007 [0.138, 0.158]
*M*. MG03 (*n* = 2)	0.352 ± 0.009 [0.346, 0.358]	0.426 ± 0.021 [0.4, 0.451]	0.126 ± 0.002 [0.124, 0.127]	0.162, 0.001 [0.162, 0.163]	0.194 ± 0.014 [0.183, 0.204]
*M. drm01* (*n* = 7)	0.455 ± 0.008 [0.442, 0.468]	0.561 ± 0.021 [0.519, 0.611]	0.113, 0.016 [0.099, 0.146]	0.277, 0.034 [0.236, 0.320]	0.213 ± 0.009 [0.197, 0.224]
*M. hanneli* (*n* = 4)	0.248 ± 0.008 [0.232, 0.267]	0.342 ± 0.009 [0.335, 0.355]	0.166 ± 0.008 [0.16, 0.176]	0.248, 0.009 [0.454, 0.492]	0.115 ± 0.001 [0.114, 0.115]
*M. madecassum* (*n* = 8)	0.94 ± 0.017 [0.913, 0.959]	1.032 ± 0.022 [1.009, 1.062]	0.201 ± 0.018 [0.188, 0.232]	0.477, 0.016 [0.454, 0.492]	0.413 ± 0.037 [0.36, 0.482]
*M. pharaonis* (*n* = 4)	0.789 ± 0.069 [0.728, 0.851]	0.786 ± 0.03 [0.756, 0.816]	0.174 ± 0.015 [0.159, 0.188]	0.376, 0.018 [0.36, 0.392]	0.385 ± 0.027 [0.363, 0.419]
*M. termitobium_*nr02 (*n* = 12)	0.383 ± 0.033 [0.313, 0.434]	0.446 ± 0.07 [0.339, 0.574]	0.158 ± 0.02 [0.105, 0.181]	0.253, 0.034 [0.201, 0.308]	0.199 ± 0.029 [0.14, 0.238]
*M. termitobium_*nr03 (*n* = 4)	0.323 ± 0.048 [0.311, 0.355]	0.358 ± 0.003 [0.355, 0.36]	0.103 ± 0.004 [0.1, 0.107]	0.207, 0.002 [0.205, 0.21]	0.152 ± 0.011 [0.145, 0.168]
*M. termitobium_*nr03b (*n* = 3)	0.33 ± 0.022 [0.311, 0.355]	0.348 ± 0.013 [0.335, 0.36]	0.11 ± 0.013 [0.098, 0.125]	0.203, 0.006 [0.199, 0.211]	0.144 ± 0.005 [0.141, 0.15]
*S*. MG01 (*n* = 2)	0.319 ± 0.022 [0.315, 0.323]	0.364 ± 0.013 [0.36, 0.368]	0.097 ± 0.007 [0.092, 0.103]	0.191, 0.001 [0.19, 0.192]	0.222 ± 0 [0.222, 0.223]
*S. fisheri* (*n* = 6)	0.509 ± 0.04 [0.458, 0.555]	0.564 ± 0.041 [0.53, 0.622]	0.17 ± 0.022 [0.138, 0.193]	0.35, 0.019 [0.328, 0.376]	0.328 ± 0.029 [0.289, 0.376]
*S. hildebrandti_*nr01 (*n* = 6)	0.274 ± 0.013 [0.252, 0.291]	0.37 ± 0.012 [0.352, 0.382]	0.113 ± 0.014 [0.101, 0.142]	0.23, 0.009 [0.214, 0.24]	0.235 ± 0.018 [0.216, 0.258]
*S. hildebrandti_*nr02 (*n* = 6)	0.318 ± 0.011 [0.322, 0.378]	0.329 ± 0.014 [0.312, 0.348]	0.11 ± 0.006 [0.1, 0.117]	0.212, 0.008 [0.236, 0.320]	0.222 ± 0.016 [0.199, 0.248]
*S. modesta* (*n* = 6)	0.347 ± 0.02 [0.322, 0.378]	0.383 ± 0.015 [0.367, 0.41]	0.098 ± 0.009 [0.1, 0.117]	0.216, 0.014 [0.193, 0.23]	0.227 ± 0.018 [0.201, 0.252]
**Species**	**AeL**	**SeL**	**VcL**	**VcaH**	**VuL**
*M*. MG01 (*n* = 3)	0.187 ± 0.009 [0.182, 0.197]	0.154 ± 0.015 [0.139, 0.169]	0.166 ± 0.007 [0.159, 0.172]	0.088 ± 0.007 [0.082, 0.095]	0.113 ± 0.003 [0.109, 0.116]
*M*. MG02 (*n* = 7)	0.188 ± 0.013 [0.174 ± 0.208]	0.157 ± 0.003 [0.154, 0.16]	0.167 ± 0.004 [0.161, 0.171]	0.089 ± 0.008 [0.077, 0.102]	0.115 ± 0.005 [0.106, 0.123]
*M*. MG03 (*n* = 2)	0.229 ± 0.037 [0.203, 0.255]	0.17 ± 0 [0.17, 0.17]	0.177 ± 0.024 [0.16, 0.195]	0.107 ± 0.007 [0.103, 0.112]	0.14 ± 0.01 [0.133, 0.147]
*M. drm01* (*n* = 7)	0.235 ± 0.009 [0.221, 0.246]	0.233 ± 0.013 [0.216, 0.255]	0.232 ± 0.007 [[0.222, 0.246]	0.133 ± 0.016 [0.118, 0.158]	0.15, 0.004 [0.145, 0.156]
*M. hanneli* (*n* = 4)	0.187 ± 0.002 [0.185, 0.188]	0.106 ± 0.004 [0.102, 0.11]	0.131 ± 0.003 [0.126, 0.134]	0.091 ± 0.005 [0.085, 0.097]	0.092 ± 0.002 [0.091, 0.095]
*M. madecassum* (*n* = 8)	0.5 ± 0.034 [0.447, 0.554]	0.35 ± 0.012 [0.329, 0.369]	0.388 ± 0.029 [0.343, 0.422	0.204 ± 0.024 [0.174, 0.24]	0.267 ± 0.026 [0.217, 0.306]
*M. pharaonis* (*n* = 4)	0.527 ± 0.047 [0.488, 0.593]	0.335 ± 0.022 [0.314, 0.365]	0.366 ± 0.028 [0.337, 0.401]	0.178 ± 0.01 [0.165, 0.187]	0.255 ± 0.011 [0.241, 0.266]
*M. termitobium_*nr02 (*n* = 12)	0.207 ± 0.031 [0.148, 0.242]	0.191 ± 0.021 [0.15, 0.21]	0.195 ± 0.03 [0.135, 0.229]	0.106 ± 0.015 [0.084, 0.132]	0.135 ± 0.017 [0.109, 0.165]
*M. termitobium_*nr03 (*n* = 4)	0.178 ± 0.01 [0.167, 0.188]	0.151 ± 0.01 [0.138, 0.159]	0.151 ± 0.006 [0.144, 0.157]	0.076 ± 0.006 [0.071, 0.084]	0.111 ± 0.006 [0.106, 0.117]
*M. termitobium_*nr03b (*n* = 3)	0.164 ± 0.008 [0.155, 0.17]	0.152 ± 0.003 [0.149, 0.154]	0.151, 0.002 [0.149, 0.152]	0.097 ± 0.005 [0.093, 0.103]	0.112 ± 0.005 [0.107, 0.116]
*S*. MG01 (*n* = 2)	0.368 ± 0.012 [0.36, 0.377]	0.382 ± 0.02 [0.368, 0.396]	0.286, 0.019 [0.272, 0.3]	0.154, 0.007 0.149, 0.158]	0.087 ± 0.01 [0.08, 0.094]
*S. fisheri* (*n* = 6)	0.49 ± 0.045 [0.432, 0.566]	0.495 ± 0.033 [0.468, 0.555]	0.391 ± 0.021 [0.369, 0.426]	0.134 ± 0.015 [0.113, 0.15]	0.142 ± 0.018 [0.121, 0.173]
*S. hildebrandti_*nr01 (*n* = 6)	0.305 ± 0.023 [0.285, 0.342]	0.313 ± 0.012 [0.296, 0.333]	0.263 ± 0.009 [0.255, 0.278]	0.091 ± 0.01 [0.074, 0.101]	0.093 ± 0.019 [0.078, 0.124]
*S. hildebrandti_*nr02 (*n* = 6)	0.281 ± 0.018 [0.254, 0.298]	0.293 ± 0.01 [0.283, 0.31]	0.249 ± 0.012 [0.239, 0.265]	0.087 ± 0.005 [0.082, 0.097]	0.088 ± 0.007 [0.075, 0.093]
*S. modesta* (*n* = 6)	0.37 ± 0.013 [0.351, 0.389]	0.375 ± 0.011 [0.366, 0.395]	0.292 ± 0.014 [0.282, 0.317]	0.129 ± 0.011 [0.112, 0.144]	0.089 ± 0.007 [0.075, 0.095]

## Data Availability

The Excel files containing the data and scripts are openly available at https://github.com/Nomenaras/Morphometrics (accessed on 8 July 2024).

## Supplementary Materials

The following supporting information can be downloaded at: https://www.mdpi.com/article/10.3390/insects15080605/s1, Table S1. Specimen information about *Monomorium* and *Syllophopsis* was used in this study. N: number of samples. “**” in the species column are valid species, “*” were morphospecies similar to a valid species, and normal/without asterisk were morphospecies distinct from known species, Table S2. Repeatability scores (R) from the Intraclass Correlation Coefficient (ICC), Table S3. Removal of allometry via regression model. Paramere height (PaH) as the independent variable, the coefficient x, and intercept are provided. Removal of Allometric Variance of shape variables was performed for the assumption of each individual having a paramere size of PaH = 0.417 mm.
