# Supplementary material for: Linear Morphometry of Male Genitalia Distinguishes the Ant Genera Monomorium and Syllophopsis (Hymenoptera: Formicidae) in Madagascar
Source: Insects. 2024 Aug 11;15(8):605. doi: 10.3390/insects15080605 (PMC11354313; doi:10.3390/insects15080605)
Supplement: Supplementary file 1 [file insects-15-00605-s001.zip › insects-3121288-supplementary.pdf]

Linear morphometry of male genitalia distinguishes the ant genera *Monomorium* and *Syllophopsis* (Hymenoptera: Formicidae) in Madagascar

Nomena F. Rasoarimalala[1,2*], Tanjona Ramiadantsoa[1], Jean Claude Rakotonirina[1, 2], Brian L. Fisher[1,3]

[1]Madagascar Biodiversity Center, Parc Botanique et Zoologique de Tsimbazaza, Antananarivo 101, Madagascar
[2]Mention Entomologie Cultures Élevage et Santé, Faculté des Sciences, Université d'Antananarivo, Antananarivo 101, Madagascar
[3]Department of Entomology, California Academy of Sciences, San Francisco, CA 94118, USA

Supplementary Materials

Table S1. Specimen information about *Monomorium* and *Syllophopsis* was used in this study. N: number of samples. "**" in the species column are valid species, "*" were morphospecies similar to a valid species, and normal/without asterisk were morphospecies distinct from known species.
Table S2. Repeatability scores (R) from the Intraclass Correlation Coefficient (ICC).
Table S3. Removal of allometry via regression model. Paramere height (PaH) as the independent variable, the coefficient x, and intercept are provided. Removal of Allometric Variance of shape variables was performed for the assumption of each individual having a paramere size of PaH = 0.417 mm.

Table S1. Specimen information about *Monomorium* and *Syllophopsis* was used in this study. N: number of samples. "**" in the species column are valid species, "*" were morphospecies similar to a valid species, and normal/without asterisk were morphospecies distinct from known species.

Specimen Code	Collection Code	Locality	Latitude	Longitude	Elevation (m)
CASENT0244570-0244572-Res	MG-57-15	Madagascar	-13.169	49.700	90
CASENT0245576-0245579-Res	MG-61-06	Madagascar	-15.157	47.734	97
CASENT0244604-0244606-Res	MG-57-17	Madagascar	-13.1695	49.700	90
CASENT0169313-0169316-Res	MG-55-13	Madagascar	-25.030	44.691	86
CASENT0265578-0265579-Res	MG-58-08	Madagascar	-13.250	49.616	182
CASENT0231784-0231786-Res	MG-58-14	Madagascar	-13.250	49.616	182
CASENT0169626-0169627-Res	MG-56-05	Madagascar	-20.067	46.999	1700
CASENT0245756-0245759-Res	MG-59-18	Madagascar	-25.030	44.691	30
CASENT0266569-0266573-Res	MG-55-04	Madagascar	-13.079	49.902	86
CASENT0433391-Res	BLF4232	Madagascar	-13.8	48.166	600
CASENT0192676-0192679-Res	BLF18284-02	Madagascar	-20.068	44.659	30
CASENT0477002-Res	BLF3257	Madagascar	-19.45	44.833	50
CASENT0229061-Res	MG-27-17	Madagascar	-18.198	47.281	701
CASENT0169895-Res	MG-50B-25	Madagascar	-23.440	43.899	46
CASENT0266008-0266010-Res	MG-09B-267	Madagascar	-21.250	47.407	1128
CASENT0245971-0245972-Res	MG-07-20	Madagascar	-12.333	49.25	360
CASENT0205372-Res	MG-08B-19	Madagascar	-18.887	47.512	1025

Specimen Code	Genus	species	N
CASENT0244570-0244572-Res	*Monomorium*	*termitobium*_nr02*	12
CASENT0245576-0245579-Res	*Monomorium*	*drm01 **	7
CASENT0244604-0244606-Res	*Monomorium*	*termitobium*_nr03*	4
CASENT0169313-0169316-Res	*Monomorium*	*pharaonis* (Linnaeus 1758)**	4
CASENT0265578-0265579-Res	*Monomorium*	MG01	3
CASENT0231784-0231786-Res	*Monomorium*	MG02	7
CASENT0169626-0169627-Res	*Monomorium*	MG03	2
CASENT0245756-0245759-Res	*Monomorium*	*termitobium*_nr03b*	3
CASENT0266569-0266573-Res	*Monomorium*	*madecassum* (Forel 1892)**	8
CASENT0433391-Res	*Monomorium*	*hanneli (Forel 1907)***	1
CASENT0192676-0192679-Res	*Monomorium*	*hanneli (Forel 1907)***	2
CASENT0477002-Res	*Monomorium*	*hanneli (Forel 1907)***	1
CASENT0229061-Res	*Syllophopsis*	*hildebrandti*_nr01*	6
CASENT0169895-Res	*Syllophopsis*	*hildebrandti*_nr02*	6
CASENT0266008-0266010-Res	*Syllophopsis*	*fisheri* (Heterick 2006)**	6
CASENT0245971-0245972-Res	*Syllophopsis*	MG01	2

CASENT0205372-Res	*Syllophopsis*	*modesta* (Santschi 1914)*	6

Table S2. Repeatability scores (R) from the Intraclass Correlation Coefficient (ICC)

Character	Definition	R *(Monomorium)*	R (*Syllophopsis)*
PaH	Paramere height	0.999	0.999
PaL	Paramere length	0.999	0.998
TeH	Telomere height	0.995	0.996
VoL	Volsella length	0.999	0.996
AeH	Valviceps height	0.998	0.994
AeL	Pienisvalvae length	0.998	0.998
SeL	Valviceps denticles length	0.999	0.999
VcL	Valviceps length	0.998	0.996
VcaH	Apical height of valviceps	0.996	0.995
VuL	Valvura length	0.998	0.955

Table S3. Removal of allometry via regression model. Paramere height (PaH) as the independent variable, the coefficient x, and intercept are provided. Removal of Allometric Variance of shape variables was performed for the assumption of each individual having a paramere size of PaH = 0.417 mm.

Character	Slope (PaH)	Intercept
PaL	-0.188	1.225
TeH	-0.305	0.470
VoL	-0.354	0.797
AeH	-0.232	0.653
AeL	-0.359	0.872
SeL	-0.540	0.886
VcL	-0.383	0.769
VcaH	-0.163	0.358
VuL	-0.066	0.349
